# Pseudonectria keratitis—emerging pathogenic fungi in the eye

**DOI:** 10.1186/s12941-024-00723-1

**Published:** 2024-07-18

**Authors:** Yongze Zhu, Peng Nan, Zhongliang Zhu, Youqi Ji, Bingqian Zhuo, Wei Xu, Yumei Ge

**Affiliations:** 1 Laboratory Medicine Center, Department of Clinical Laboratory, Zhejiang Provincial People’s Hospital (Affiliated People’s Hospital), Hangzhou Medical College, Hangzhou, Zhejiang 310014 China; 2 Key Laboratory of Precision Medicine for Head and Neck Cancers of Zhejiang Province, Zhejiang Provincial People’s Hospital, Hangzhou, Zhejiang 310014 China

**Keywords:** Fungal keratitis, Matrix-assisted laser desorption/ionization time-of-flight mass spectrometry, ITS sequencing, *Pseudonectria foliicola*, Early identification

## Abstract

**Background:**

Infectious keratitis, a significant contributor to blindness, with fungal keratitis accounting for nearly half of cases, poses a formidable diagnostic and therapeutic challenge due to its delayed clinical presentation, prolonged culture times, and the limited availability of effective antifungal medications. Furthermore, infections caused by rare fungal strains warrant equal attention in the management of this condition.

**Case presentation:**

A case of fungal keratitis was presented, where corneal scraping material culture yielded pink colonies. Lactophenol cotton blue staining revealed distinctive spore formation consistent with the *Fusarium species*. Further analysis using Matrix-Assisted Laser Desorption/Ionization Time-of-Flight Mass Spectrometry (MALDI-TOF MS) identified the causative agent as *Fusarium proliferatum*. However, definitive diagnosis of *Pseudonectria foliicola* infection was confirmed through ITS sequencing. The patient’s recovery was achieved with a combination therapy of voriconazole eye drops and itraconazole systemic treatment.

**Conclusion:**

*Pseudonectria foliicola* is a plant pathogenic bacterium that has never been reported in human infections before. Therefore, ophthalmologists should consider *Pseudonectria foliicola* as a possible cause of fungal keratitis, as early identification and timely treatment can help improve vision in most eyes.

## Introduction

Corneal inflammation caused by various factors is collectively referred to as keratitis. Infection is the most common cause of keratitis, especially when the host is associated with damage or loss of corneal epithelial cells or the body’s resistance is reduced [1]. Infectious keratitis is widely recognized as a leading contributor to preventable visual impairment and blindness globally [2], and was formally designated as a neglected tropical disease by the World Health Organization in 2019 [3]. Among them, fungal keratitis (FK) is a serious corneal disease that poses a threat to public health and economy. In recent years, the incidence rate of FK has soared due to the increase in the number of people with immune deficiency or basic diseases such as diabetes and chronic eye diseases, as well as the widespread use of contact lenses [4]. According to the survey, fungal infection is the leading cause of infectious keratitis worldwide, accounting for approximately 50% of all infectious keratitis, and is the second most common blinding eye disease after cataracts [2, 5].

The common pathogenic fungi for FK include *Aspergillus spp.*, *Fusarium spp.*, *Penicillium spp.*, and *Candida spp.* [6, 7]. However, in recent years, an increasing number of rare FK cases have been reported [8], which poses great challenges for the diagnosis and treatment of FK. The clinical characteristics of FK are very similar to bacterial keratitis and are often difficult to distinguish. This often delays diagnosis, leading to a poor prognosis and even the development of endophthalmitis [9]. Emerging fungal pathogens and resistance to existing antifungal drugs further contribute to the poor prognosis of FK. Therefore, keratitis caused by fungal infection should not be ignored [10]. Here, we report a case of keratitis caused by *Pseudonectria foliicola* infection. This is a plant pathogenic fungus that can cause wilt disease in boxwood, belonging to the Ascomycota phylum and Ascomycota family, often spreading in soil [11]. It has never been reported in human infections before.

## Case presentation

### Clinical features

**Fig. 1:**
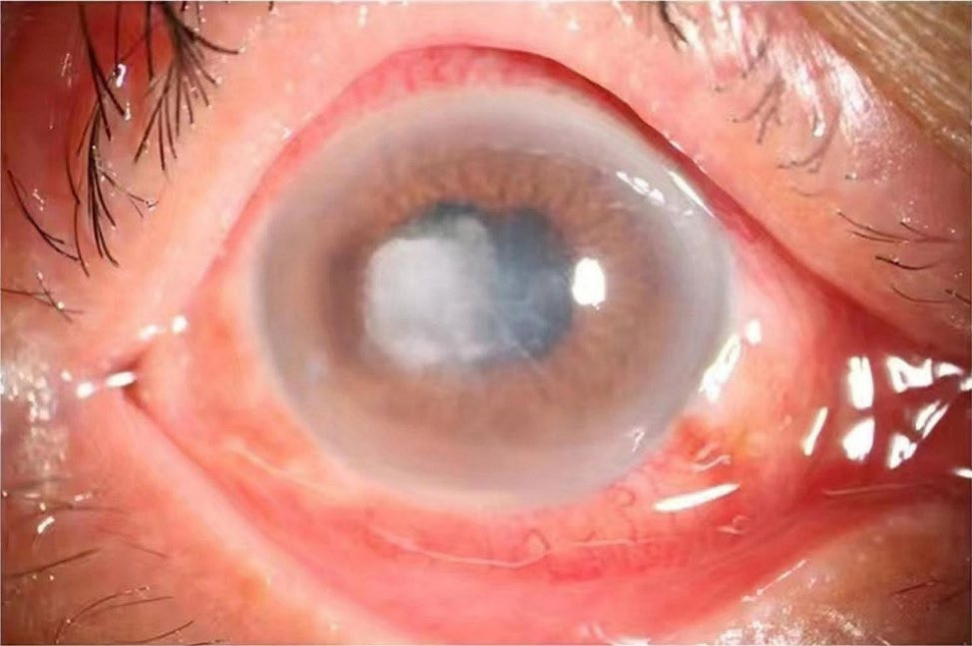
The patient’s eye image clearly shows a white mist like object inside the eye

A 60s female patient sustained an injury to her right eye while engaged in agricultural activities, resulting in foreign body sensation, ocular redness, pain, reduced vision, eyelid swelling, and tearing. She initially sought treatment at a local hospital, where she received fluometholone and levofloxacin eye drops, along with oral cefprozil, but experienced no significant improvement. Consequently, she was referred to our hospital for further evaluation and management. Upon examination, the patient displayed mild eyelid swelling, with prominent conjunctival congestion. A white, slightly elevated, circular lesion, approximately 4 mm × 3 mm in size, was observed on the central cornea, temporal to the limbus. The lesion’s boundaries were indistinct, and its surface was slightly dry, with a noticeable central endothelial fold. The anterior chamber was deep, revealing a pus accumulation of approximately 1.5 mm beneath. The iris was fully adherent to the pupil, with clear texture, a round shape of 3 mm x 3 mm, and an absent light reflex. The lens showed minimal opacity (Fig. 1). Routine peripheral blood tests revealed no abnormal findings.

### Etiological examination

**Fig. 2:**
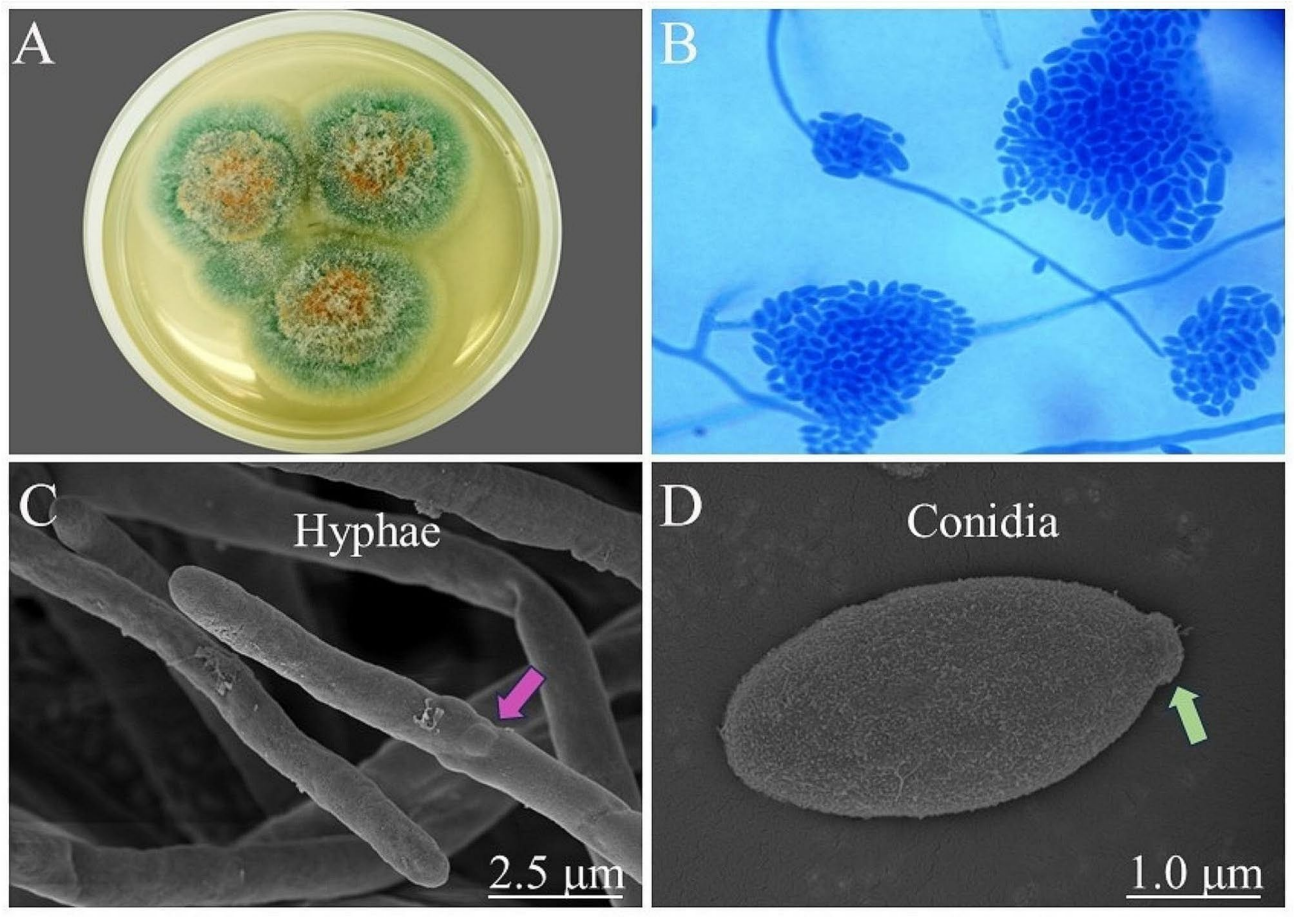
Morphological identification of *Pseudonectria foliicola*. (**A**) *Pseudonectria foliicola* colony culture with yellow-green mycelium and pink spores. (**B**) Lactophenol cotton blue staining demonstrated characteristic sporulation similar to *Fusarium spp.*, aseptate hyphae branched at acute angles with numerous fusiform to ellipsoidal conidia in different size. (**C** and **D**) The conidiophore was ellipsoidal and cuticular roughness with a bud scar (purple arrow) at one end, approximately 20 μm in length (figure E). Characteristic protuberant sites (green arrow) for germinating solitary blastospore and annellide were noted on the sidewall surface of hyphae

**Fig. 3:**
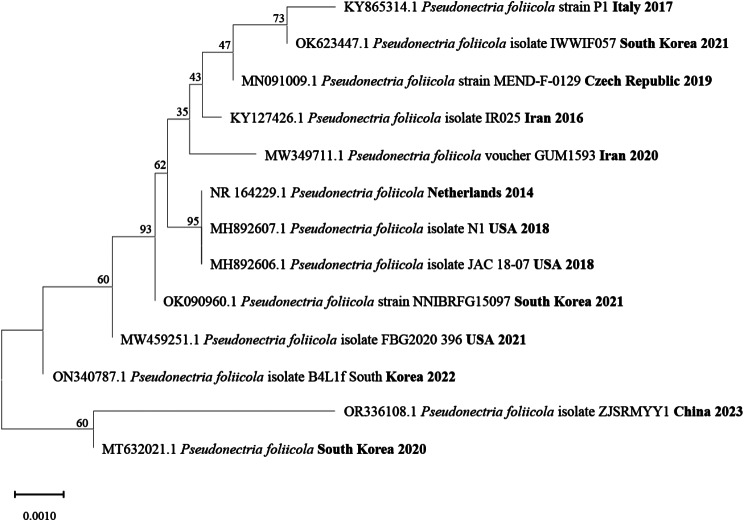
Phylogenetic tree of *Pseudonectria foliicola* ZJSRMYY1

Take the patient’s corneal scraping material for fungal culture. After 7 days in a constant temperature incubator at 28℃, yellow green filamentous fungal colonies grow, producing a large amount of spores with a pink color in the middle of the colonies (Fig. 2A). Lactophenol cotton blue staining showed characteristic spore formation similar to that of the *Fusarium spp.* (no septal hyphae branching at acute angles, with many spindle to oval shaped conidia of different sizes) (Fig. 2B). *Pseudonectria foliicola* was confirmed by sequencing and BLAST analysis of internal transcribed spacer DNA regions (GenBank accession number OR336108.1) (Fig. 3). The scanning electron microscope showed the ultrastructure of the fungus. The conidia are elliptical in shape, with rough epidermis and bud marks at one end, approximately 20 μm. On the sidewall surface of the mycelium, characteristic protrusions of germinating individual spores and segments were observed (Fig. 2C and D). It is worth mentioning that in the early stages of diagnosis, we used matrix assisted laser destruction ionization time of flight mass spectroscopy (MALDI-TOF MS) for identification. However, the identification results showed *Fusarium proliferatum*, which caused some misdiagnosis in our diagnosis.

### Treatment and outcome

After admission, considering that the patient’s lesion was caused by a plant injury, combined with the patient’s current eye examination and medical history, we suspected that fungal corneal ulcers were highly likely. Therefore, the patient was given antifungal treatment with voriconazole eye drops once an hour and levofloxacin eye drops five times a day to prevent infection. At the same time, the patient was given oral itraconazole for systemic antifungal treatment with 0.4 g for the first day, 0.3 g for the second day until corneal ulcer scars appeared and then reduced 0.2 g per day. Five days later, the patient’s symptoms improved significantly. The patient was allowed to be discharged with medication and instructed to continue follow-up treatment.

## Discussion

The incidence of FK varies greatly throughout the world, and FK is of particular concern in developing countries and tropical and subtropical regions due to the growth habits of the fungus [12]. Despite their clinical manifestation similarities with bacterial keratitis, the pathogenesis of FK differs [13]. In short, the pathogenesis of FK is that the infected fungus is able to survive and grow in the cornea, while being able to penetrate the host’s defense mechanisms to reach the internal tissues, digest and absorb the host’s nutrients, and resist the attack of the host’s immune system [14]. Epidemiological studies reveal a higher incidence in males, with a 1.6:1 ratio, potentially linked to higher agricultural occupations, which expose individuals to plant-related trauma, a primary cause of FK [15–17]. Other risk factors include contact lens use, pre-existing ocular conditions, skin fungal infections, prolonged antibiotic or steroid use, and a history of eye surgery [18]. Clinicians should be vigilant for these factors when suspecting FK, as exemplified by our case where a patient, injured by plants during farming, was initially misdiagnosed and treated inappropriately due to the overlooked predisposing factor at a lower healthcare facility.

Early diagnosis is crucial for the treatment of FK. The traditional diagnostic method is to collect corneal scraping material for fungal culture, which is also the main basis for diagnosing FK. However, invasive tissue sampling carries a certain risk of complications, and cultivation usually takes a long time, which can delay the condition and increase the burden on patients [19]. Rapid staining microscopy is a fast, simple, and inexpensive detection method, but it depends on the work experience of inspectors [20]. In vivo confocal microscopy (IVCM) is a new instrument used in recent years for the early diagnosis and guidance of treatment of fungal corneal ulcers, which can dynamically observe the morphology of fungal hyphae in the cornea [21]. Compared to traditional detection methods, IVCM can achieve non-invasive detection while achieving the same sensitivity and specificity [22], but it has not broken through the limitations of species identification. MALDI-TOF MS is currently the most commonly used pathogens identification method in hospital microbiology departments. This method is convenient, fast, and can identify the vast majority of pathogens. But MALDI-TOF MS is also limited by the database, and for rare pathogens, this method may also result in identification failure or errors. In our testing process, MALDI-TOF MS mistakenly identified the pathogen as *Fusarium proliferatum*, which greatly misled the diagnosis. Finally, we confirmed keratitis caused by *Pseudonectria foliicola* through ITS sequencing. DNA sequencing is the gold standard for microbial identification, which not only distinguishes species and identifies clinically rare species, but also helps predict antifungal drug sensitivity [23]. Therefore, when facing rare pathogens infections, we should pay more attention to sequencing.

*Pseudonectria foliicola*, the major pathogen causing the leaf blight of boxwood, is widely distributed globally and further expands with the distribution of boxwood [11]. However, the eye lesions in our patients came from straw, a genus of gramineous rice, indicating further expansion of *Pseudonectria foliicola*. *Pseudonectria foliicola* is characterized by the production of a large number of pink fungal spores on the foliage surfaces of infected plants [24], which is why we suspected the MALDI-TOF MS result and further ITS sequencing was conducted.

Due to poor eye penetration and low bioavailability of currently available antifungal drugs to the expected site of action, the treatment of FK is challenging [25]. The first line of treatment for FK starts with local antifungal drugs, while surgical treatment is not the first choice [26]. Due to the lack of previous reports of human infection with *Pseudonectria foliicola*, and considering the patient’s age and heavy surgical burden, we chose to use voriconazole eye drops for local treatment. Afterwards, based on monitoring the patient’s liver function, we administered the first dose of 0.4 g of itraconazole to the patient and gradually reduced the dosage until the patient’s corneal ulcer scarring occurred. Under such combined treatment, patients recover.

## Conclusion

We first illustrated the possibility of this emerging pathogenic fungus infecting humans and spreading across species. Therefore, ophthalmologists should consider including *Pseudonectria foliicola* as a possible cause of fungus keratitis, as early identification and prompt management helped majority of the eyes to improve visual acuity. In addition, when facing rare pathogens infections, we should pay more attention to the sequencing results to avoid misdiagnosis caused by their rarity.

## Data Availability

No datasets were generated or analysed during the current study.
